# Interpretation of clinically meaningful change in cancer palliative care patients’ quality of life: minimally important difference for EORTC QLQ-C15-PAL

**DOI:** 10.1186/s41687-025-00858-5

**Published:** 2025-03-19

**Authors:** Kikuko Miyazaki, Yoshimi Suzukamo, Masayuki Ikenaga, Shozo Ohsumi, Mari Saito, Eriko Satomi, Kojiro Shimozuma, Takeo Nakayama

**Affiliations:** 1 Department of Health Informative, School of Public Health, Kyoto University, Yoshida-Konoe cho, Sakyo-ku, Kyoto, 606-8501 Japan; 2 Comprehensive Unit for Health Economic Evidence Review and Decision Support, Ritsumeikan University, Kyoto, Japan; 3 Palliative Medicine, Yodogawa Christian Hospital, Osaka, Japan; 4 Department of Brest Surgery, National Hospital Organization Shikoku Cancer Center, Matsuyama, Japan; 5 Department of Palliative Care, Yokohama Municipal Citizen’s Hospital, Yokohama, Japan; 6 Department of Palliative Care, National Cancer Center Hospital, Tokyo, Japan; 7 Department of Biomedical Science, Ritsumeikan University, College of Life Sciences, Shiga, Japan

## Abstract

**Background:**

Palliative care for cancer helps improve and maintain patients’ quality of life (QOL). Clinically meaningful changes in QOL measures are helpful when considering how a patient would want to spend the final days of their life. This study aimed to estimate the minimally important differences (MIDs) for within-person change for the European Organisation for Research and Treatment of Cancer QOL Questionnaire Core 15 Palliative Care (EORTC QLQ-C15-PAL) domains in advanced cancer patients in palliative care.

**Method:**

Participants in this multicenter observational study comprised patients with advanced cancer receiving palliative care in the last year before death. The EORTC QLQ-C15-PAL was administered at two-week intervals. During the second assessment, patients also completed the Global Rating of Change (GRC) scale to collect their subjective assessments of changes in their condition since the first assessment. MID for QOL score with a correlation of 0.3 or more with GRC score changes were estimated using anchor- and distribution-based methods.

**Results:**

Among the 257 screened patients at 13 facilities, we analyzed 181 (92 male; mean age: 67). The mean survival time was 131 days. Notably, the number of patients who responded “no change” for the GRC items was large (63–128). Anchor-based MIDs differed depending on the change direction (improvement vs. deterioration). The MIDs for meaningful within-person change may be used in clinical practice.

**Conclusion:**

We estimated the MIDs in EORTC QLQ-C15-PAL in patients with advanced cancer with a life expectancy of less than one year, both anchor- and distribution-based.

## Introduction

Palliative care for cancer aims to improve and maintain a patient’s quality of life (QOL). According to the World Health Organisation (WHO), “palliative care is an approach that improves the QOL of patients and their families (adults and children) who are facing problems associated with life-threatening illness” [1]. In patients with advanced cancer, QOL is the principal endpoint [2]. However, few clinical studies of QOL in palliative care patients can be found in general medical journals. Performing QOL assessments with palliative care patients is difficult due to the deterioration of a person’s physical and cognitive states at the end of life [3]. While QOL assessments are necessary for palliative care, it is also essential to consider ways to reduce the patient burden as much as possible [4]. Therefore, several QOL questionnaires have been developed that consider the characteristics of palliative care patients [4–6]. One of them is the 15-item European Organization for Research and Treatment of Cancer QOL Questionnaire Core15 Palliative Care (EORTC QLQ-C15-PAL), which was developed to assess the QOL of palliative care patients. It aims to reduce the burden associated with collecting self-report data from patients with advanced diseases and a limited life expectancy. The survey is designed to be completed quickly by these patients. This questionnaire is a shortened version of the European Organisation for Research and Treatment of Cancer Quality of Life Questionnaire Core 30 (EORTC QLQ-C30) [7], which is widely used in clinical cancer research [8, 9].

To facilitate the use of the EORTC QLQ-C15-PAL in palliative care practice with a target group of patients with a predicted life expectancy of several months, it is necessary to estimate the minimally important differences (MIDs). MIDs are used to interpret meaningful changes in clinical QOL findings. The literature includes numerous terms that refer to the same concept as the MID, including the Subjective Significance Questionnaire (SSQ), Minimal Clinically Important Difference (MCID), Clinically Important Difference (CID), Minimally Detectable Difference (MDD), and Minimal Important Change (MIC) [10–15]. However, we use terminology from the International Society for QOL Research (ISOQOL) dictionary published in 2015. MCID, MID, MIC, Responsiveness to Change, Sensitivity to Change, and Smallest Detectable are listed together in the “change” entry. Within this broad meaning, we use MID as the minor meaningful change in the QOL score of advanced cancer patients receiving palliative care at two time points within that group. The group-level MID is used in sample size calculations [10] for studies involving between-group differences, such as in clinical trials. Recently, a distinction is made between within-patient changes and between-group differences [16]. This study focused on the smallest change in QOL scores that was a meaningful within-person change [17]. Our primary objective was to understand and use changes in palliative care patient QOL assessments in healthcare settings where providers work directly with patients.

Numerous studies have been conducted on MIDs to interpret changes in scores on the EORTC QLQ-C30, which is used as a disease-specific scale for cancer [10, 18–24]. It has been pointed out that MIDs for QOL scores differ depending on the setting and sample being studied, so they are not strict reference values [25]. Studies of MIDs for the EORTC QLQ-C15-PAL have already been conducted using samples of patients with bone metastases and brain metastases undergoing palliative radiotherapy [26, 27]. The purpose of our study is the MID estimation of the EORTC QLQ-C15-PAL domain scores to utilize QOL assessment for daily clinical practice and decision-making considerations in patients with advanced cancer receiving usual palliative care and life expectancy of less than one year.

## Methods

### Design

An observational study was designed to address this purpose.

### Participants

Although palliative care is needed for people with non-cancer life-threatening illnesses, we have focused in this paper on patients with advanced cancer and a life expectancy of less than one year. Participants were recruited at facilities practicing palliative care, including hospital palliative care wards, hospice care in-patient and out-patient facilities, and facilities involving palliative care consultation teams and palliative home care.

Inclusion criteria were that the patient had a physician’s prognosis of less than one year of life expectancy and had already received palliative treatment. Candidates were excluded if they were younger than 20 or 80 or older and if a physician determined they could not fill the QOL questionnaire themselves due to physical or cognitive impairment.

### Measures

A baseline QOL assessment was conducted at the start of palliative care, and a second assessment was performed two weeks later, along with a patient self-reported survey regarding the degree of change in QOL during that period. Subsequently, follow-up was conducted until the day of the participant’s death. The Japanese version of the EORTC QLQ-C15-PAL, validated in Japanese, was used for QOL assessment [28, 29]. The degree of self-reported change in QOL was investigated using the Global Rating of Change (GRC) scale.

The EORTC QLQ-C15-PAL was developed by reducing the number of questions from 30 to 15 on the EORTC QLQ-C30, the most widely used QOL assessment for cancer patients. Since symptoms of palliative care patients vary, the EORTC QLQ-C15-PAL does not cover all physical symptoms, and the development of a module to cover more symptoms has been reported [30]. However, for patients with advanced cancer, the ability to measure QOL continuously from cancer treatment is a unique feature. The EORTC QLQ-C15-PAL is recommended for use by patients with advanced incurable cancer who are expected to live only a few months. The recall period is one week. The questionnaire encompasses ten domains: physical functioning (PF; three items) and emotional functioning (EF; two items); pain (PA; two items), fatigue (FA; two items); five single-item symptoms: dyspnoea (DY), insomnia (SL), nausea and vomiting (NV), appetite loss (AP), and constipation (CO); one question about global health or QOL (QL). Items are rated on a four-point scale, except for the QL question, which is rated on a seven-point scale. All scales and individual items are converted to a score from 0‒100 [11]. Thus, high scores on a functional scale denote better functioning, while high scores for a symptom scale or item denote more of that symptom (i.e., a worse condition).

The GRC has ten items corresponding to the ten domains of the EORTC QLQ-C15-PAL. Participants were asked to rate the degree of change in each domain on a seven-point scale: large deterioration, deterioration, small deterioration, no change, small improvement, improvement, and large improvement. The question wording for each of the ten domains was agreed upon by four university researchers from the fields of palliative care research (KM), QOL measurement methods (YS), QOL assessment (KS), and epidemiology (TN) using the Delphi Method.

Patients who met the eligibility criteria were asked to complete the EORTC QLQ-C15-PAL. We asked them to retake the EORTC QLQ-C15-PAL and complete the GRC two weeks later. For the single-item symptoms of NV, DY, IS, AP, and CO, patients were instructed to check “no change” on the GRC if they did not have any of those symptoms. We referred to physician records for clinical information, such as patient profile, Eastern Cooperative Oncology Group (ECOG) performance status (PS), primary disease, reasons for missing data, and date of death

### Analysis

We calculated the scores for the first and second QOL assessments [9]. Subsequently, we obtained the frequency distribution for the seven response categories from “large deterioration” to “large improvement.” For the anchor-based approach, MIDs were determined by calculating the means for the degree of improved and deteriorated change reported for each of the ten GRC items. The polychoric correlation coefficient was used to confirm the strength of the association between change in EORTC QLQ-C15-PAL and GRC scores for each of the ten domains. If the correlation was less than 0.3, we excluded the item from any further MID analysis because an anchor with a very low correlation to the change should not be used to determine whether a significant change has occurred [30]. Our anchor-based estimates of MID are calculated from the categories of ‘small improvement’ and ‘small deterioration’. For the distribution-based approach, we calculated the MID values for each of the EORTC QLQ-C15-PAL scales using a 0.5 standard deviation. Finally, we compared each MID with the smallest possible in response scores for the corresponding EORTC QLQ-C15-PAL [9] scale. SPSS version 25 was used to perform the statistical analyses.

## Results

### Participants

**Fig. 1:**
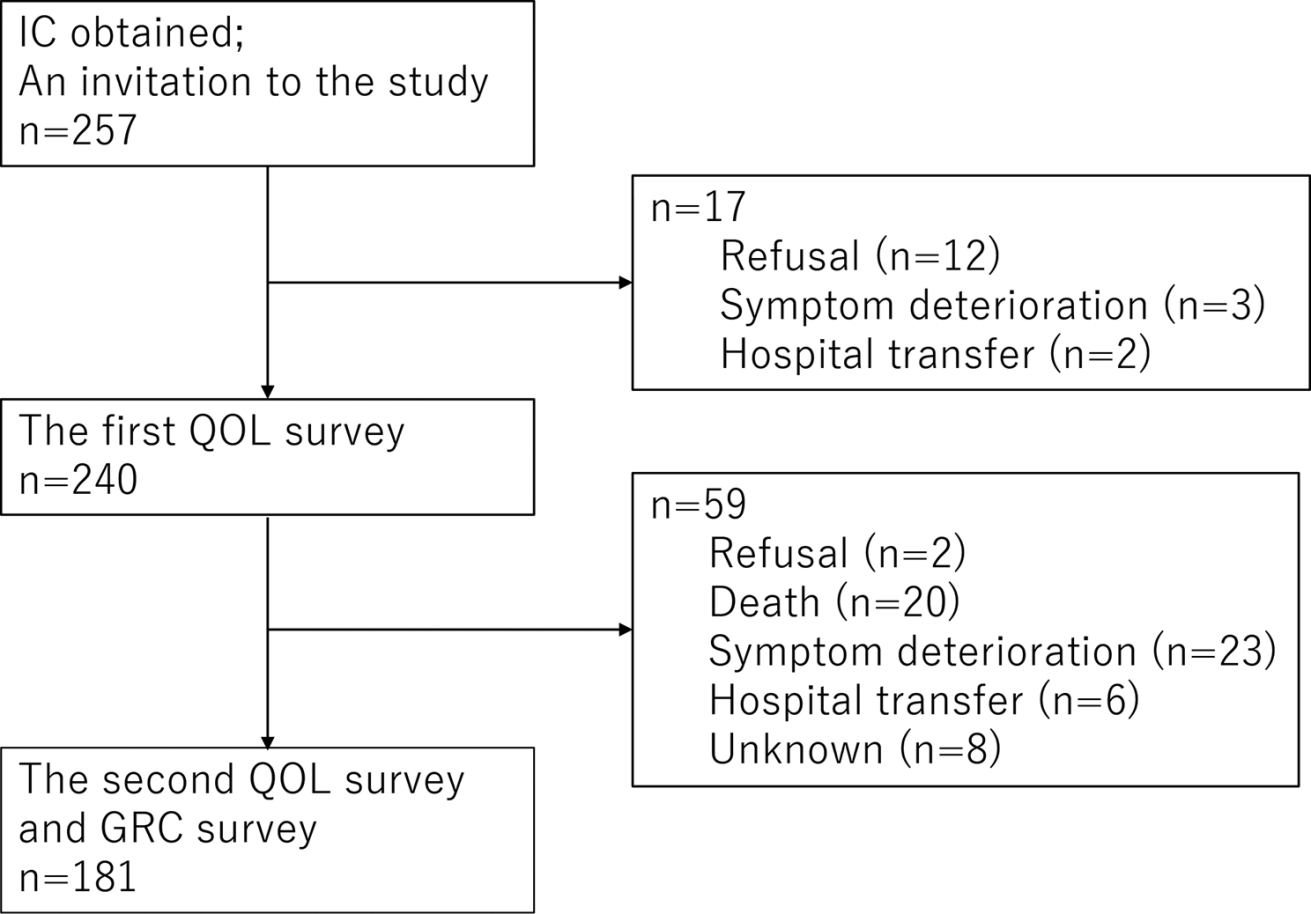
Participant selection

In Japan, the Cancer Control Act of 2007 established palliative care at cancer centers nationwide. Thus, regional disparities in palliative care are decreasing. This study’s participants were from provincial and metropolitan cities, including cancer centers, university hospitals, city hospitals, and their linked home care facilities. The flowchart of the participants is shown in Fig. 1. The study was explained to 257 palliative care patients at 13 participating facilities. Of these, 240 patients consented to participate and completed the QOL assessment themselves. Of these 240 patients, 181 patients (92 male, 89 female, mean age: 67; standard deviation [SD]: 12) answered the second assessment. The largest number of participants was 67 (37%) with ECOG PS3 (Capable of only limited self-care, confined to bed or chair more than 50% of waking hours). The most common primary diseases in participants included lung cancer in 31 (17%), colon cancer in 24 (13%), breast cancer in 21 (12%), and prostate cancer in 14 (8%). The mean survival time was 131 days (SD: 144). Regarding settings, 66 patients (36% of the sample) were in palliative care wards in hospitals, 97 (54%) were receiving interventions by palliative care consultation teams, and the remaining 18 (10%) were receiving palliative care at home (Table 1).

**Table 1:** Participant characteristics (n = 181)

Gender	Male	92
	Female	89
Age	Mean (SD)	67 (12)
ECOG PS	0	10
	1	30
	2	61
	3	67
	4	13
Primary Cancer	Lung cancer	31
	Colon cancer	24
	Brest cancer	21
	Prostate cancer	14
	Stomach cancer	12
	Oesophageal cancer	10
	Cervical cancer	10
	Pancreatic cancer	9
	Renal cancer	8
	Liver cancer	7
	Other	35
Day until death	Mean (SD)	131 (144)
Setting	Hospice/Palliative care	66
	Palliative care team concertation	97
	Home care	18
City/country scale	Metropolitan city	110
	Provincial city	71
Palliative chemotherapy	Yes	88
	No	88

### Measurements

**Fig. 2:**
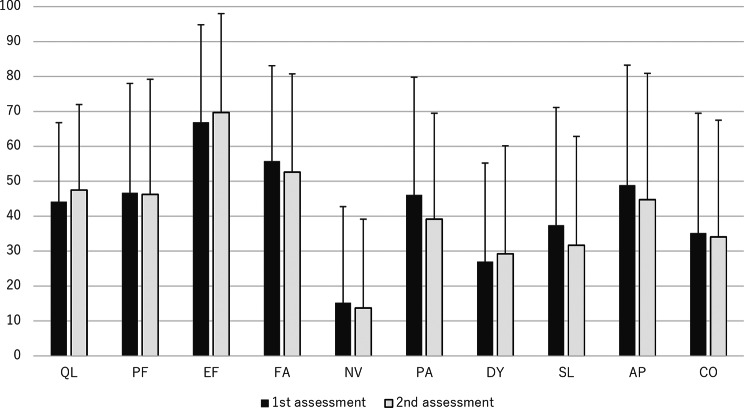
EORTC QLQ-C15-PAL results

Results from the EORTC QLQ-C15-PAL assessments at two weeks showed that, compared to baseline scores, the mean DY score had deteriorated slightly while all other mean domain scores (QL, PF, EF, FA, PA, NV, SL, AP, and CO) improved slightly. However, none of the observed mean changes were statistically significant (Fig. 2).

The distribution of responses to the 10 GRC items, the anchor for the anchor-based approach, showed that the frequency of the response “no change” for the symptoms in the QOL questionnaire ranged from 83 (46%) for FA to 128(71%) for NV. For functioning, it was 63(35%) for PF and 87(48%) for EF. The frequency of the response “small improvement” for the symptoms ranged from 11(6%) of the respondents for NV to 37(20%) for PA. For functioning, the frequencies were 41(23%) for PF and 31(17%) for EF. The frequency of “small deterioration” for the symptoms ranged from 17 (9%) for NV to 39 (22%) for FA. For functioning, frequencies were 25 (14%) for PF and 27 (15%) for EF (Table 2).

**Table 2:** GRC response distribution

	n	Lage deterioration	Deterioration	Small deterioration	No change	Small improvement	Improvement	Lage improvement
		n (%)	n (%)	n (%)	n (%)	n (%)	n (%)	n (%)
QL	179	6 (3%)	16 (9%)	27 (15%)	77 (43%)	33 (18%)	17 (9%)	3 (2%)
PF	179	10 (6%)	24 (13%)	25 (14%)	63 (35%)	41 (23%)	15 (8%)	1 (1%)
EF	177	6 (3%)	9 (5%)	27 (15%)	87 (48%)	31 (17%)	15 (8%)	2 (1%)
FA	180	6 (3%)	18 (10%)	39 (22%)	83 (46%)	24 (13%)	9 (5%)	1 (1%)
NV^a^	178	6 (3%)	7 (4%)	17 (9%)	128 (71%)	11 (6%)	7 (4%)	2 (1%)
PA	180	5 (3%)	17 (9%)	18 (10%)	85 (47%)	37 (20%)	15 (8%)	3 (2%)
DY^a^	179	7 (4%)	8 (4%)	22 (12%)	122 (67%)	14 (8%)	5 (3%)	1 (1%)
IS^a^	180	2 (1%)	7 (4%)	24 (13%)	106 (59%)	28 (16%)	8 (4%)	5 (3%)
AP^a^	181	8 (4%)	17 (9%)	24 (13%)	93 (51%)	24 (13%)	9 (5%)	4 (2%)
CO*	179	8 (4%)	5 (3%)	21 (12%)	111 (61%)	26 (14%)	7 (4%)	1 (1%)

^a^ Subjects were instructed to select “Unchanged” for an item if they had no symptoms for the item

The correlation coefficient between the GRC score and change in QOL score by domain were all greater than 0.3 [31]; hence, MID estimation was performed for all domains of the EORTC QLQ-C15-PAL (Table 3).

**Table 3:** Correlation between GRC score and change in QOL score by item/domain

Item/Domain	Coefficient	***p***
QL	0.468	< 0.001
PF	0.499	< 0.001
EF	0.328	< 0.001
FA	−0.351	< 0.001
PA	−0.483	< 0.001
NV	−0.584	< 0.001
DY	−0.443	< 0.001
SL	−0.417	< 0.001
AP	−0.498	< 0.001
CO	−0.528	< 0.001

An anchor-based approach was used to determine meaningful within-person change MIDs. Using the GRC as the anchor resulted in different values depending on whether the patient perceived their condition had improved or deteriorated. The MID for small improvement on the QL and functional scales ranged from 4.9 to 12.1, and the MID for small deterioration ranged from − 1.2 to − 8.0. The MID for small improvement on the symptom scale ranged from − 23.8 to 4.8, and the MID for small deterioration ranged from 4.6 to 27.5. For the distribution-based approach, MIDs were estimated at 0.5 standard deviations, from 11.7 to 18.4 (Table 4).

**Table 4:** MIDs using anchor-based and distribution-based approaches (n = 181)

	Anchor-based						Distribution-based (change)
	Small improvement	(n)	No change	(n)	Small deterioration	(n)	0.5SD
QL	12.1	(33)	1.5	(77)	−1.2	(27)	12.4
PF	4.9	(41)	3.5	(63)	−3.3	(25)	11.7
EF	9.4	(31)	4.6	(87)	−8.0	(27)	14.1
FA	−16.2	(24)	−8.1	(83)	6.4	(39)	15.3
NV	−1.7	(11)	−5.5	(128)	27.5	(17)	14.7
PA	−14.9	(37)	−5.7	(85)	4.6	(18)	15.5
DY	−4.8	(14)	−3.6	(122)	16.7	(22)	14.2
SL	−23.8	(28)	−5.0	(106)	12.5	(24)	17.3
AP	−16.7	(24)	−7.3	(93)	7.2	(24)	18.4
CO	−20.5	(26)	−3.0	(111)	19.0	(21)	17.7

## Discussion

This study estimated the MID for meaningful within-person changes in EORTC QLQ-C15-PAL domain score changes over two weeks in patients with advanced cancer receiving palliative care and a life expectancy of less than one year. Compared to the 0.5 SD of the distribution-based results (range: 11.7–18.4 for distribution-based estimates; Table 4), the MID estimates from the anchor-based method are highly variable (range: − 23.8–12.1 for anchor-based estimates of improvement; Table4). Furthermore, the range of estimated MID values indicating an improvement or deterioration (Table 4) deserve thoughtful consideration.

Values estimated using an anchor-based approach studies often differ depending on the kind of anchor used. Moreover, Wyrwich et al. [32] argued that triangulation should be used to derive an MID threshold by examining several sources of evidence of meaningful change. In our study of advanced cancer patients, only the QL domain’s distribution-based estimate (12.4) demonstrated near agreement with the anchor-based estimate to improvement (12.1), while none of the other domain score MID estimates domains exhibit a noteworthy similarity between the anchor-based and distribution-based approaches. If only these two sources of evidence are available to examine meaningful change over time, the anchor-based estimates in Table 4, each incorporating the patient-reported change information in the respective 10 GRC items, provide a more informed estimate than the distribution-based estimates in our study.

Our reported findings compare and contrast with the MID estimates reported in two 2016 EORTC QLQ-C15-PAL MID investigations led by Ramen et al. and Berard et al. [26, 27] using anchor- and distribution-based methods. It is important to emphasize that the patients participating in these two prior palliative cancer studies had generally better health than the patients in our study of advanced cancer patients. Using the Karnofsky Performance Status (KPS) and ECOG PS scales to compare participants’ health status across the studies, the mean KPS in Raman et al. [26] was 76.9 and 70.7 in Bedard et al. [27]. In our study, 37% of the participants had ECOG PS 3 (corresponding to 30% to 40% of KPS), 34% had PS 2 (corresponding to 50% to 60% of KPS), 16% had PS 1 (corresponding to 70% to 80 % of KPS), 7% had PS 4 (corresponding to 20% to 10% of KPS), and 5 % had PS 0 (corresponding to 90% to 100% of KPS) [33]. With known limitations on the use of MIDs for QOL scores outside of a specific patient population [25, 31] or cancer type [21], a notable reason for differences between our EORTC QLQ-C15-PAL MID estimates and the MID estimates reported in Raman et al. and Bedard et al. for cancer patients receiving palliative care is likely due to these baseline differences in the patients’ health/performance statuses.

Interestingly, our distribution-based MID estimates (range: 11.7–18.4; Table 4) closely resembled the anchor-based and distribution-based MID range observed in Raman et al. (range: 11.4–17.2)[26]. However, it is important to note that the Raman et al. study [26] used of the EORTC-QLQ-C15-PAL’s overall QOL scale (QL) response, a single-item domain, as the anchor item for MID estimation versus the 10 patient-reported GRC items that anchored our study’s respective anchor-based results, which demonstrated greater variation across domains in the resulting anchor-based MID estimates when compared to Raman et al. [26].

Indeed, our study anchor-based results showed a wide range of MID estimates across the EORTC QLQ-C15-PAL domains. Importantly, for nearly all reported patient groups (Table 4), this study’s anchor-based MID estimates for the pain score were larger for improvement (−14.9) and smaller for deterioration (4.6), which is both important and consistent with the anchor-based pain domain results in the 2016 PAL MID studies (i.e., deterioration 6.2, improvement − 23.6 in Raman et al. and deterioration − 17.3, improvement − 15.6 in Bedard et al.) [26, 27]. Despite the important differences in participants’ health/performance status, these three studies’ MID estimates of the pain scale may continue to indicate that cancer patients in palliative care are sensitive to slight worsening of pain but do not feel better without significant pain relief/improvement. This trend is intriguing, and hopefully can be future investigated in future studies in this critical cancer patient population.

The normal distribution of GRC scores for most domains suggests that palliative care intervention has potentially contributed to some patients with advanced cancer remaining stable or even showing improvement. This observation is contrary to the general expectation that the palliative patient’s condition would deteriorate. Nonetheless, the large number of “no change” GRC responses in this study may reflect the short period (2 weeks) between the first and second survey or patient dropout at follow up due to deteriorating health (Fig. 1). Nonetheless, in clinical palliative care, when QOL assessments are used as reference data for decision-making and advance care planning for terminally ill patients, global changes reported in GRC item responses are crucial knowledge. QOL often deteriorates more gradually versus improvements in terminally ill patients. When we examined patients’ self-reported degree of change in status for anchor items, our MID estimates demonstrated differences depending on whether the patient was improving or worsening in the distribution of meaningful change in the GRC [35].

In palliative care clinical practice, a vital score changes of great important occurs when the patient deteriorates. Therefore, the within-person MID estimates in Table 4 need to be further interpreted for application in individual patients assessed over time. If individual patient responses on the EORTC QLQ-C15-PAL questionnaire deteriorate two categories in the NV domain and one category in the other domains, a meaningful within-person change has likely occurred, based on the results in this study. The score difference for a one-category change in the 0–100 NV domain only would be 16.7; for a two-category change, it would be 50 [9]. The anchor-based deteriorated MID estimate is 27.5; this means the individual score change is between one category (16.7) and two categories (50) shift. For the other symptom domains (PA, DY, SL, AP, CO), the score changes by 33.3 on the 0–100 scale when the response on the EORTC QLQ-C15-PAL questionnaire deteriorates by one category/response. In other words, the anchor-based deteriorated MID domain score estimates (PA: 4.6, DY: 16.7, SL: 12.5, AP: 7.2, CO: 19.0) were smaller than the one-category change in score (33.3). The deteriorated MID for function domains (QL: − 1.2, PF: − 3.3, EF: − 8.0) were also smaller than the one-category change in QOL score (QL:16.7, PF:6.7, EF:8.3) [9]. Thus, a one-category change in the direction of deterioration in domains other than NV also changed a meaningful within-person change.

Studies of clinical importance thresholds have shown high sensitivity and specificity in identifying clinically essential symptoms and functional health impairments [35]. The estimated results of the EORTC QLQ-C15-PAL as an average of the MIDs may be used in clinical practice as timely information that contributes to the preparation, review, and decision-making in the ever-changing situations that occur in the final stages of life while taking individual circumstances into account.

This study has some limitations. First, because of the prevalence of “no change” responses on the GRC anchor items, the number of responses used for the MID analyses of improvement and deterioration was small. Numerous other studies using the anchor have considered this a barrier to estimating MID values. As noted in this study, some patients did not experience the single-item symptoms assessed in the EORTC QLQ-C15-PAL domains. In those cases, the patients were instructed to check “no change” as their respective GRC response, contributing to the notable use of this response option. Investigating how to best interpret the response of “no change” is a topic for future research. Second, we used the patient’s GRC responses as an anchor. However, as observed by Ousmen et al. [36], we did not test the reliability or the validity of these anchor items, and full validation of the GRC items as the response anchor is a future challenge.

## Conclusions

This study estimated the two-week MID in EORTC QLQ-C15-PAL domain scores in patients with advanced cancer with a life expectancy of less than one year, using both anchor-based and distribution-based. These MID estimates have practical use for interpreting QOL assessments in this important patient population.

## Data Availability

The datasets used and analyzed during the current study are available from the corresponding author upon reasonable request.

## Abbreviations

EORTC QLQ-C15-PAL: The European Organisation for Research and Treatment of Cancer Quality of Life Questionnaire Core 15 Palliative Care
MID: Minimally important differences
QOL: Quality of life
GRC: Global Rating of Change
WHO: The World Health Organisation
EORTC QLQ-C30: The European Organisation for Research and Treatment of Cancer Quality of Life Questionnaire Core 30
ISOQOL: The International Society for Quality of Life Research
PF: Physical functioning
EF: Emotional functioning
PA: Pain
FA: Fatigue
DY: Dyspnea
SL: Insomnia
NV: Nausea and vomiting
AP: Appetite loss
CO: Constipation
QL: Global health/Quality of life
ECOG PS: Eastern Cooperative Oncology Group (ECOG) performance status
KPS: Karnofsky Performance Status
